# No going back? Reversibility and why it matters for deep brain stimulation

**DOI:** 10.1136/medethics-2018-105139

**Published:** 2019-01-10

**Authors:** Jonathan Pugh

**Keywords:** deep brain stimulation, neuroethics, psychosurgery, autonomy, psychiatry

## Abstract

Deep brain stimulation (DBS) is frequently described as a ‘reversible’ medical treatment, and the reversibility of DBS is often cited as an important reason for preferring it to brain lesioning procedures as a last resort treatment modality for patients suffering from treatment-refractory conditions. Despite its widespread acceptance, the claim that DBS is reversible has recently come under attack. Critics have pointed out that data are beginning to suggest that there can be non-stimulation-dependent effects of DBS. Furthermore, we lack long-term data about other potential irreversible effects of neuromodulation. This has considerable normative implications for comparisons of DBS and brain lesioning procedures. Indeed, Devan Stahl and colleagues have recently argued that psychiatric DBS should be subject to the same legal safeguards as other forms of psychosurgery, supporting their position by forcibly criticising the claim that DBS is reversible. In this paper, I respond to these criticisms by first clarifying the descriptive and evaluative elements of the reversibility claim that supporters of DBS might invoke, and the different senses of ‘reversibility’ that we might employ in discussing the effects of medical procedures. I go on to suggest that it is possible to defend a nuanced version of the reversibility claim. To do so, I explain how DBS has some effects that are stimulation dependent in the short term, and argue that these effects can have significant normative implications for patient well-being and autonomy. I conclude that we should not abandon a nuanced version of the reversibility claim in the DBS debate.

Deep brain stimulation (DBS) is a neurosurgical treatment modality that is widely used in the treatment of movement disorders associated with neurological conditions such as Parkinson’s disease.^1 2^ It is also being investigated as an experimental treatment for patients suffering from a range of serious treatment-refractory psychiatric disorders such as depression,^3^ obsessive-compulsive disorder^4^ and anorexia nervosa.^5 6^ DBS poses a number of perioperative and postoperative risks^7^ that have been widely discussed elsewhere in the ethics literature.^8–10^ Yet, for many patients, the potential benefits of DBS may plausibly outweigh these risks.

In the context of Parkinson’s disease brain lesioning procedures (such as thalamotomy and pallidotomy) have been largely superseded by DBS, where both interventions are available for a particular patient. Part of the reason for this is that comparative studies have established that DBS has fewer adverse effects and results in a greater overall improvement in function for patients.^11 12^ In contrast, the  increasing interest in psychiatric DBS has been accompanied by a growing number of studies investigating lesioning procedures (such as anterior capsulotomy and anterior cingulotomy) for psychiatric disorders.^13–15^ ^i^ Crucially, to date there have been no published comparative studies of DBS and brain lesioning procedures for psychiatric disorders (unlike Parkinson’s disease).^16^

So, although DBS is often described as a ‘last resort’ treatment for some psychiatric patients who have exhausted conventional treatment avenues, this is not strictly true; brain lesioning surgery might also be posited as a further treatment modality that could offer a chance of some therapeutic benefit for some such patients.^16 17^ Moreover, given both the paucity of evidence for both interventions, and the lack of any comparative studies, we do not yet have conclusive evidence to establish that DBS would be a more effective treatment than a lesioning procedure for a particular psychiatric disorder. In the absence of such evidence, the question of whether we should understand DBS to be a preferable treatment modality to lesioning procedures in psychiatry must turn on other factors.

In particular, the following three factors are typically invoked to suggest that DBS is preferable to lesioning approaches in the psychiatric context. First, in experimental contexts, DBS allows for the investigation of different neuronal targets. This is particularly important in contexts where there is a lack of consensus concerning the neural underpinnings of the targeted disorder.^6 18^ Second, clinical teams can titrate stimulation parameters to the needs of the particular patient, allowing them to potentially reach a balance between harmful side effects and the therapeutic effect of stimulation. Third and finally, it is possible to entirely cease stimulation (and to entirely explant the DBS system if the patient is willing to undergo a further neurosurgical procedure). On the basis of this feature, DBS is frequently described as reversible, in contrast to the irreversible effects of brain lesioning procedures.

In considering whether to pursue DBS treatment or lesioning where comparative effectiveness has not been established, the above three beneficial features of DBS are to be weighed against factors speaking in favour of lesioning over DBS. These include the lower financial costs of lesioning, the lack of need for the recipient’s long-term compliance with treatment protocols, and the fact that there are perioperative risks associated with the long-term maintenance of a DBS device that are not applicable to lesioning procedures.^17 19^

Notwithstanding the support that these factors lend to lesioning approaches, as Stahl *et al* note (p 5), the apparent reversibility of DBS is often offered to justify the claim that DBS is nonetheless superior to lesioning approaches. Yet these authors (and others) have called the reversibility of DBS into question by appealing to empirical data suggesting non-stimulation-dependent effects of DBS. This has considerable ethicolegal implications. Indeed, Stahl *et al* forcibly criticise the claim that DBS is reversible in order to argue that psychiatric DBS should be subject to the same restrictive safeguards as brain lesioning procedures in psychiatry.^20^ Elsewhere, the normative significance of this debate about DBS’ reversibility is amplified by the fact that brain lesioning procedures are increasingly being championed as a rival paradigm to DBS for psychiatric disorders.^15 17^

In response to this recent trend in the literature, I shall defend what I shall call the ‘reversibility claim’. The reversibility claim, as I understand it, incorporates two elements:

1. The *descriptive* claim that DBS evinces reversible effects.
2. The *evaluative* claim that, *ceteris paribus*, DBS is a morally preferable form of intervention to brain lesioning procedures, by virtue of its reversible effects.

It is vital that both elements of the reversibility claim are recognised; I shall consider each in turn. I shall concede that critics of the reversibility claim are correct to claim that it is false if the descriptive element is understood to apply indiscriminately to all aspects of DBS, such that it is understood to evince *only* reversible effects. However, I shall argue that it is possible to defend a more plausible version of the reversibility claim.

## The descriptive claim

The descriptive element of the reversibility claim is theoretically prior to the evaluative element, in the sense that the latter only has any traction if DBS is reversible in at least some respects. Perhaps for this reason, in their critique of the reversibility claim, Stahl *et al* do not concern themselves with the evaluative question of whether we should attribute particular normative significance to reversibility per se. Instead, they attack the descriptive element of the reversibility claim, by querying whether DBS is in fact reversible. Having outlined how ‘… DBS is widely perceived to be a better substitute for classical lesioning because of its perceived reversibility and adjustability,’^20^ they go on to note:

The possibility of brain lesioning, while unintended and less severe than in other procedures, is still present as the mere insertion of the electrodes can cause irreparable tissue damage to the patient’s brain.^20^ (p 5)

Stahl *et al* are right to raise this point. Data from postmortem studies of patients who had undergone DBS in the treatment of a movement disorder have shown that lead insertion, and perhaps even chronic stimulation, can lead to a small amount of brain tissue damage, including glial scarring.^21^ There is also emerging evidence of other non-stimulation-dependent neurological effects of treatment beyond the possibility of microlesioning. For example, Diana Ruge and colleagues have explored the retention of clinical effects of DBS following the cessation of stimulation in patients undergoing DBS for movement disorders for over 4.5 years.^22 23^ They note the possibility that prolonged stimulation of this sort may produce long-term neural reorganisation underlying the persisting clinical effect.^22^ Maslen *et al* have also described a case of unexpected complications of DBS treatment for chronic pain, in which two patients developed de novo epilepsy following treatment.^24^ There is also some emerging evidence of non-stimulation-dependent psychiatric effects of DBS in depression.^25^

In addition to evidence of non-stimulation-dependent effects, Jennifer Mundale has pointed out that the absence of evidence about the long-term effects of DBS treatment should weaken the credence that we lend to the descriptive element of the reversibility claim. We have insufficient evidence to establish with certainty the truth (or falsity) of the claim that DBS is (or is not) reversible over long periods of time.^26^

In light of the above, it might appear that the reversibility claim is dead in the water, and that it should no longer be invoked in order to claim that DBS is a morally preferable treatment modality to lesioning. However, as I shall explain below, this conclusion would be too hasty. Prior to doing so, it is important to clarify some different aspects of the descriptive claim.

First, when the claim pertains to DBS *in toto*, it is unlikely to be true that DBS is wholly reversible. Reversibility in this sense is not binary; rather, the reversibility of a procedure *in toto* is likely to admit of degree, on the basis that procedures will differ with respect to both the number and significance of their reversible effects. In order to assess the degree to which an intervention is reversible, we need to assess the quality and number of its reversible effects.

In making this assessment, it is important to distinguish two senses of reversibility that we might mean to invoke. The concepts of reversibility and irreversibility may be used to track claims about the *permanence* of a state of affairs. We may say that a necessary and sufficient condition of a change from state S1 to state S2 at time *t* being ‘permanent’ is that following *t*, S2 obtains and there is ‘no going back’ to S1 after *t*. A non-permanent, or temporary change is simply one that is not fixed in this way; although S2 occurs after *t*, S1 may still be able to obtain at some later point. Notice that the permanence of an effect is a binary matter.

Of course, under this description there is one permanent change that *any* medical procedures will evince. Once one has undergone a medical procedure (at *t*), one cannot (at *t+1*) change the fact that this description about one’s past now obtains. Notably, that this fact obtains may have had important implications for one’s well-being following (t). For example, the fact that a patient began receiving a treatment 1 year ago might mean that they enjoyed a higher quality of life in the past year than they would have otherwise.

I mention this kind of permanent change only to set it aside. In thinking about the changes that DBS evinces (and whether or not they are permanent), we are not primarily interested in the trivial point that the aforementioned sort of facts about the past must obtain permanently. We should of course be mindful of such facts, and the implications that they might have for the states to which it might be possible for a patient to return once they have embarked on a treatment path. However, in this context, we are primarily interested in the *prospective* effects of DBS, effects concerning which one can sensibly ask the question ‘can this state of affairs be changed in the future?’

Crucially, there may also be a sense in which we also use the concept of reversibility to capture more than just a claim about non-permanence. In a stronger sense of reversibility, it is not sufficient that the change be temporary; it must also be the case that one is able to *control* (to some extent) when and whether S1 or S2 obtains after *t*. I shall call this sense of reversibility ‘full-blooded’ reversibility, and refer to reversibility *simpliciter* when I mean to refer to the sense of reversibility that is meant to capture only considerations of non-permanence.

On both understandings, permanent changes will always be irreversible changes (and vice versa). However, on the full-blooded understanding, there may be some temporary changes that one cannot control. To illustrate, the common cold is a temporary medical condition; if you have it, you will not typically have it for long. However, although we can use remedies to manage its symptoms to some degree, we can exert little control over when our immune system will ‘cure’ us by successfully fighting off the infection. Should we say then that the common cold is reversible? The answer depends on whether we mean to ask whether it is reversible *simpliciter*, or reversible in a full-blooded sense.

To be clear, I am not concerned with the question of which sense of reversibility is ‘correct’. Rather, I draw the distinction here because although stimulation-dependent effects of DBS can be understood as being reversible in both senses, this is not true of all of the non-stimulation-dependent effects that critics of DBS have recently discussed, as I shall now explain.

## The evaluative claim

Stahl *et al* mount a convincing attack against the descriptive element of the reversibility claim understood in a particularly strong sense. It is true that DBS *in toto* is not wholly reversible; it is not the case that it has *only* reversible effects. However, as I shall now explain, this need not be the sense that defenders of the reversibility claim mean to invoke, particularly in light of the evaluative element of the reversibility claim.

Notably, Stahl *et al* do not attend to this evaluative element of the reversibility claim. Accordingly, let me begin by illustrating the importance of drawing out both the descriptive and evaluative elements of the reversibility claim. Consider the following: Even minor superficial surgery leads to a permanent scar; it thereby has an irreversible effect. This descriptive claim is also true of an amputation surgery that causes the permanent loss of a limb. However, the mere fact that the two procedures share the property of ‘having permanent and irreversible effects’ does not imply that that we should evaluate them equivalently. The two effects clearly have considerably different implications for our evaluation of the procedure.

This raises the question of what might affect the strength of the reasons associated with different irreversible effects. Naturally, I cannot provide a full account here, but one plausible suggestion is that the strength of such reasons is significantly affected by the implications of the effect for the individual’s well-being. The permanent loss of a limb is more significant than the development of a permanent scar in part because we can expect the former to be considerably more detrimental to the individual’s well-being than the latter. Considerations of permanence have particular relevance in this regard, because the duration of a harm can feature prominently in our assessment of its impact on our well-being. Alternatively, advocates of patient autonomy might maintain that patients should determine the strength of the reasons associated with different irreversible effects.

I will return to this issue below. At this point though, I want to stress that in demonstrating that DBS is not *wholly* reversible, Stahl *et al* are arguing against a straw man. If one is to mount a telling objection to the reversibility claim, one has to engage with both its descriptive and evaluative elements. The key question is whether the irreversible features of DBS that one has identified ground comparably weighty reasons to the irreversible features of brain lesioning procedures. Notably, Stahl *et al* fail to defend such a claim.

Indeed, at least some of the irreversible effects of DBS outlined in the previous section arguably have limited implications for our evaluation of the intervention. For instance, the possibility that DBS may produce long-term neural reorganisation that could underlie a persisting clinical effect is not clearly problematic. To see why, consider that on the basis of their evidence, Ruge *et al* hypothesise that neural reorganisation may occur in patients who have received long term-stimulation of more than 4.5 years,^22^ noting that a persistent clinical effect was not similarly observed in a previous on/off study of DBS after only 6 months of treatment.^27^ ^ii^ Accordingly, even assuming that Ruge *et al*’s hypothesis is correct, the neural reorganisation they propose arises in patients following *years* of prolonged stimulation. As such, patients will have a considerable amount of time to get used to the effects of treatment and/or to cease it before this permanent, irreversible, *clinically beneficial* effect obtains.

Furthermore, brain tissue damage of the sort involved in the microlesioning that Stahl *et al* emphasise does not ground particularly weighty moral reasons per se. We have little evidence to suggest that this damage adversely affects patient well-being among the many patients who have already undergone DBS for movement disorders. However, in light of Mundale’s point highlighted above, this can only form a partial response to the problem at hand. Absence of evidence is not evidence of absence, and we do not have sufficient data about the effects of long-term brain stimulation and/or the tissue damage caused by electrode implantation to be certain about their effects on patient well-being.

Yet, with respect to the effects of tissue damage caused by electrode damage there is scope for challenging the claim that the observed effects are truly permanent. The little data that we have are equivocal about the permanence of such changes. Although there is evidence that beneficial implantation effects in some patients can be maintained without stimulation (as described above), there is also evidence to suggest that some clinical effects of implantation-associated lesioning typically abate shortly after implantation.^26^ So, even if there are some effects of implantation-associated lesioning, it is not clear that these effects will be permanent. However, while this may entail that the effects may be reversible simpliciter, this does not entail that the changes are reversible in the full-blooded sense. In this regard, we may note that implantation-associated lesioning effects that are reversible in this way would still differ from stimulation-dependent effects of DBS, which are in contrast reversible in the full-blooded sense.

Below, I shall explain why full-blooded reversibility can have normative significance beyond reversibility *simpliciter*. At this point though, it should be clear that the above observations should lead defenders of DBS to temper the overly broad descriptive claim that ‘DBS is reversible.’ However, they should not lead us to abandon a nuanced version of the reversibility claim. The reason for this is that we have a great deal of evidence from the use of DBS in movement disorders to suggest that both important therapeutic effects and also unintended side effects of the procedure, across a range of targets, can bestimulation dependent in the short term. This is quite compatible with the claim that DBS may have some permanent and irreversible features, and the fact that we lack *long-term* evidence regarding the reversibility of changes following chronic stimulation, both in terms of reversibility *simpliciter* and ‘full-blooded reversibility’.

Accordingly, I do not believe that Stahl *et al* discredit the descriptive element of the reversibility claim that is actually operative in the most plausible account of how DBS differs from lesioning procedures. But what can be said in favour of the evaluative claim (which Stahl *et al* do not address) that features of DBS that are stimulation dependent in the short term should carry normative significance?

Again, considerations of well-being and autonomy plausibly play an explanatory role here. Let me begin by discussing the implications of non-permanence, which features in both reversibility *simpliciter* and ‘full-blooded’ reversibility. As I suggested above, with respect to a harm’s duration it seems straightforwardly true, *ceteris paribus*, that a permanent harm is worse than a temporary harm, at least when considered in isolation. However, the prudential calculation in comparing DBS and brain lesioning procedures is more complex than simply weighing temporary harms against permanent harms. The reason for this is that we must also trade these features off against the weighting of the potentially permanent benefits of lesioning, compared with the temporary (although repeatable in a continuous manner) benefits promised by DBS. While permanent harms are clearly worse than temporary harms *ceteris paribus*, the permanent benefit afforded by a therapeutically effective lesioning procedure would be better than the temporary benefit of an equally therapeutically effective DBS intervention. After all, the therapeutic effect of lesioning would be achieved without the prudential cost of the long-term maintenance of a DBS device and its attendant risks. With respect to well-being alone then, the reversibility of DBS may not speak wholly in its favour.

Considerations of autonomy arguably lend stronger support to the claim that the reversible nature of DBS’ effects is normatively significant, particularly given their ‘full-blooded’ reversibility. At the outset, it should be acknowledged that there are a number of obstacles to obtaining valid informed consent to *either* DBS or lesioning procedures, particularly in the psychiatric context. Due to the experimental nature of the procedures in this context, clinical teams will typically only be able to provide limited information about the potential risks and benefits of the procedures. Moreover, candidate patients may have borderline decision-making capacity, and they are likely to be in a position of considerable vulnerability, given the intractable nature of their condition.^28 29^

However, the fact that many stimulation-dependent effects of DBS are reversible *simpliciter* and ‘full-blooded’ reversible means that DBS facilitates the exercise of patient autonomy in a number of ways that lesioning procedures cannot. First, the reversibility of DBS in this sense allows for greater opportunities for patients to make autonomous decisions about the course of their treatment. Unlike lesioning procedures, DBS is not a one-off intervention, but rather an ongoing therapeutic process requiring repeated instances of stimulation. By virtue of this, the patient is able to exercise autonomous choice diachronically in a way that the recipient of lesioning procedure cannot. If she chooses to undergo DBS, she is not forever committed to its effects; rather, she can gain a deeper understanding of how the treatment affects her, and choose whether the effects in question are ones that she wishes to support by continuing treatment, and when to reinitiate or cease these effects. This is a particularly important feature of DBS when considering its application in disorders where the perceived absence of control plays a significant role in the targeted psychopathology.^30^

Furthermore, this feature of DBS also allows patients to realise more of their informed preferences. To illustrate, suppose treatment X leads a patient to develop a foreseen but unintended adverse side-effect. A patient who had been willing to consent to undergo X on the basis of their understanding of the risks of the side-effect associated with X prior to treatment may also have been counterfactually *unwilling* to undergo treatment if she had known that the foreseeable but unintended side effect in question would *in fact* occur. Where X and its side effects are reversible in a full-blooded sense, this has the straightforward implication for autonomy that the patient’s preference to avoid these unwanted effects in such a scenario is actionable.

A final way in which the reversibility of stimulation-dependent effects of DBS facilitates autonomy is that it provides a way in which to overcome a significant obstacle to obtaining valid consent to any intervention that radically ameliorates an otherwise treatment refractory disorder. Any such medical intervention is plausibly an example of what Laurie Paul has described as a ‘transformative experience’. A transformative experience is one that is both radically new to the agent and that also changes her in a fundamental way.^31^ Both brain lesioning procedures and DBS can plausibly be transformative in this sense. A much-discussed case study of DBS (in Parkinson’s disease) described by Leentjens *et al* provides a compelling example.^32^

In this case, a patient undergoing DBS for Parkinson’s disease began to experience a stimulation-dependent manic state that rendered him mentally incompetent. Although this abated when stimulation was ceased, ceasing stimulation exacerbated his motor impairment. Accordingly, the patient could choose either (A) to cease stimulation and to be admitted to a nursing home because of physical invalidity, but retaining their mental competence, or (B) to continue stimulation and to be admitted to a psychiatric ward because of their manic state, but with acceptable motor capacity. Crucially, because the patient’s stimulation-dependent manic state could be reversed, the patient retained capacity to make his own treatment decision on this matter when not under stimulation. The patient chose to continue stimulation.

The inducement of a manic state is plausibly an example of a transformative experience. It is radically new in the sense that it may lead one to engage in uncharacteristic and erratic behaviours, as well as exhibiting different, unintelligible, moods. Of course, it is rare for a patient to face this sort of dichotomous choice between mental capacity and physical impairment. Nonetheless, unexpected complications of these procedures do occur, sometimes with unpredictable effects for the individual patient; and as this case illustrates, such experiences can appropriately be construed as transformative.

However, ethical issues related to transformative experiences in DBS are not restricted to rare and striking cases. A more common transformative adverse effect of these interventions might be the so-called ‘burden of normality’ that they may impose on patients, a burden typified by adverse psychological, behavioural, affective and sociological effects.^33 34^ The ‘burden of normality’ refers to the difficulties that some patients may experience when they receive effective treatment, particularly for a chronic condition that may have been at the forefront of their lives for a long period. The effects of this burden following neurosurgical interventions detailed by Gilbert can deeply affect patients’ emotional lives, values and relationships in ways that may be radically new to them, particularly if they have been chronic sufferers of a psychiatric disease.^34^

The fact that neurosurgical interventions may elicit a transformative experience raises further problems for patient autonomy beyond those outlined above. A defining feature of a transformative experience is that the agent lacks epistemic access to what it will be like to undergo that experience.^31^ Accordingly, if autonomous decision-making requires substantial understanding of one’s options, it is unclear whether one can autonomously decide to undergo a treatment that we believe will elicit a transformative experience. Moreover, such an experience might serve to change the very values that undergird autonomous decision-making; this possibility is particularly salient in the psychiatric context, where the aim of treatment may be to alter the patient’s evaluative stance.

If one accepts that neurosurgical interventions can elicit transformative experiences, then there is a further way in which the full-blooded reversibility of DBS has normative significance. Such reversibility matters because it allows an avenue for circumventing this obstacle to consenting to treatments that we believe might elicit a transformative experience. Unlike lesioning procedures, DBS can grant the recipient some degree of epistemic access to what it will be like to undergo a transformative experience, without permanently committing them to that state of being. Moreover, the patient can control when and whether the changed state obtains. Consider the Leentjens *et al*’s case. In this case, the patient decided to continue DBS treatment having been granted epistemic access both to what it was like to suffer from severe motor impairment, and also what it was like to suffer from a manic state without that motor impairment. A procedure that was not reversible in this manner would not allow the patient such an opportunity; the choice to undergo the procedure would commit the patient to that unknown and radically changed state of being. DBS allows for this opportunity, and for the patient to control when and whether the changed state obtains.

## Conclusion

Advocates of DBS have been too loose in their descriptions of the intervention as reversible *simpliciter*, and critics are quite right to object to this. Moreover, both sides of the debate should agree on the need to further investigate the neural effects of long-term stimulation, particularly at a time when DBS is being considered as a treatment for psychiatric diseases that typically have a far earlier onset than the movement disorders for which the procedure is more commonly used.

However, dispensing with the reversibility claim on this basis would be to throw the baby out with the bathwater. DBS can have some effects, both good and bad, which are stimulation dependent in the short term, and therefore reversible in that period. Moreover, the kinds of effect in question can bear significant weight in the prudential cost-benefit analysis of treatment. Indeed, the fact that the effects of a medical intervention are reversible in the short term may make the difference between a patient being willing to consent to treatment and a patient refusing treatment.

We should not abandon a version of the reversibility claim that is more sensitive to empirical data, and which distinguishes the normative significance of different reversible features, and different forms of reversibility. Of course, establishing this version of the reversibility claim is not alone sufficient to establish that DBS is a preferable treatment modality to lesioning procedures. The *ceteris paribus* clause is hugely important here. Such an argument would require attending to further considerations of safety, efficacy, cost-effectiveness and various patient selection criteria that might be relevant for different disorders and different neural targets. However, the reversibility that DBS does exhibit can be of considerable moral significance. We should thus not abandon the reversibility claim without strong countervailing empirical evidence and moral arguments.

## Footnotes

i
Although, for some criticisms of Liu’s study, see ref 30.

ii
Ruge *et al* caveat this by noting that (1) their patients might have behaved differently compared with Tisch *et al*’s group; and that (2) since Tisch *et al* did not study patients after 48 hours withdrawal from DBS, it cannot be ruled out that the immediate adverse clinical effects of turning DBS off (observed in Tisch *et al*’s study) may not have persisted beyond that period.

## References

[1] Deuschl G, Schade-Brittinger C, Krack P, et al A randomized trial of deep-brain stimulation for Parkinson’s disease. N Engl J Med 2006;355:896–908. 10.1056/NEJMoa060281 16943402

[2] Rodriguez-Oroz MC, Obeso JA, Lang AE, et al Bilateral deep brain stimulation in Parkinson’s disease: a multicentre study with 4 years follow-up. Brain 2005;128:2240–9. 10.1093/brain/awh571 15975946

[3] Bergfeld IO, Mantione M, Hoogendoorn ML, et al Deep brain stimulation of the ventral anterior limb of the internal capsule for treatment-resistant depression: a randomized clinical trial. JAMA Psychiatry 2016;73:456–64. 10.1001/jamapsychiatry.2016.0152 27049915

[4] Denys D, Mantione M, Figee M, et al Deep brain stimulation of the nucleus accumbens for treatment-refractory obsessive-compulsive disorder. Arch Gen Psychiatry 2010;67:1061–8. 10.1001/archgenpsychiatry.2010.122 20921122

[5] Lipsman N, Lam E, Volpini M, et al Deep brain stimulation of the subcallosal cingulate for treatment-refractory anorexia nervosa: 1 year follow-up of an open-label trial. Lancet Psychiatry 2017;4:285–94. 10.1016/S2215-0366(17)30076-7 28238701

[6] Park RJ, Scaife JC, Aziz TZ Study protocol: using deep-brain stimulation, multimodal neuroimaging and neuroethics to understand and treat severe enduring anorexia Nervosa. Front Psychiatry 2018;9 10.3389/fpsyt.2018.00024 PMC5898619 29681866

[7] Grill WM Safety considerations for deep brain stimulation: review and analysis. Expert Rev Med Devices 2005;2:409–20. 10.1586/17434440.2.4.409 16293080

[8] Schermer M Ethical issues in deep brain stimulation. Front Integr Neurosci 2011;5 10.3389/fnint.2011.00017 PMC3096836 21625629

[9] Clausen J Ethical brain stimulation - neuroethics of deep brain stimulation in research and clinical practice. Eur J Neurosci 2010;32:1152–62. 10.1111/j.1460-9568.2010.07421.x 21039955

[10] Lipsman N, Glannon W Brain, mind and machine: what are the implications of deep brain stimulation for perceptions of personal identity, agency and free will? Bioethics 2013;27:465–70. 10.1111/j.1467-8519.2012.01978.x 22681593

[11] Schuurman PR, Bosch DA, Bossuyt PM, et al A comparison of continuous thalamic stimulation and thalamotomy for suppression of severe tremor. N Engl J Med 2000;342:461–8. 10.1056/NEJM200002173420703 10675426

[12] Esselink RA, de Bie RM, de Haan RJ, et al Unilateral pallidotomy versus bilateral subthalamic nucleus stimulation in PD: a randomized trial. Neurology 2004;62:201–7. 10.1212/01.WNL.0000103235.12621.C3 14745054

[13] D’Astous M, Cottin S, Roy M, et al Bilateral stereotactic anterior capsulotomy for obsessive-compulsive disorder: long-term follow-up. J Neurol Neurosurg Psychiatry 2013;84:1208–13. 10.1136/jnnp-2012-303826 23733922

[14] Christmas D, Eljamel MS, Butler S, et al Long term outcome of thermal anterior capsulotomy for chronic, treatment refractory depression. J Neurol Neurosurg Psychiatry 2011;82:594–600. 10.1136/jnnp.2010.217901 21172856

[15] Liu W, Li D, Sun F, et al Long-term follow-up study of mri-guided bilateral anterior capsulotomy in patients with refractory anorexia Nervosa. Neurosurgery 2018;83:86–92. 10.1093/neuros/nyx366 28945886

[16] Hariz M, Hariz G-M Therapeutic Stimulation Versus Ablation. in Handbook of Clinical Neurology: Brain Stimulation. 116: Elsevier, 2013:63–71. 10.1016/B978-0-444-53497-2.00006-1 24112885

[17] Müller S, Riedmüller R, van Oosterhout A Rivaling paradigms in psychiatric neurosurgery: adjustability versus quick fix versus minimal-invasiveness. Front Integr Neurosci 2015;9:27 10.3389/fnint.2015.00027 25883557 PMC4383041

[18] Park RJ, Singh I, Pike AC, et al Deep brain stimulation in anorexia nervosa: hope for the hopeless or exploitation of the vulnerable? the oxford neuroethics gold standard framework. Front Psychiatry 2017;8 10.3389/fpsyt.2017.00044 PMC5357647 28373849

[19] Hariz MI, Hariz GM : Lozano AM, Hallett M, Therapeutic Stimulation Versus Ablation. in Brain Stimulation. 116: Elsevier.

[20] Stahl D, Cabrera L, Gibb T Should DBS for psychiatric disorders be considered a form of psychosurgery? ethical and legal considerations. Sci Eng Ethics 2018;24:1119–42. 10.1007/s11948-017-9934-y 28653164

[21] Grill WM Signal considerations for chronically implanted electrodes for brain interfacing. 2008. 21204401

[22] Ruge D, Cif L, Limousin P, et al Shaping reversibility? Long-term deep brain stimulation in dystonia: the relationship between effects on electrophysiology and clinical symptoms. Brain 2011;134:2106–15. 10.1093/brain/awr122 21705425

[23] Ruge D, Cif L, Limousin P, et al Longterm deep brain stimulation withdrawal: clinical stability despite electrophysiological instability. J Neurol Sci 2014;342:197–9. 10.1016/j.jns.2014.05.011 24857354

[24] Maslen H, Cheeran B, Pugh J, et al Unexpected complications of novel deep brain stimulation treatments: ethical issues and clinical recommendations. Neuromodulation 2018;21:135–43. 10.1111/ner.12613 28557242 PMC5811790

[25] Gilbert F Deep brain stimulation for treatment resistant depression: postoperative feelings of self-estrangement, suicide attempt and impulsive–aggressive behaviours. Neuroethics 2013;6:473–81. 10.1007/s12152-013-9178-8

[26] Mundale J Reversibility and deep brain stimulation. Journal of Cognition and Neuroethics 2016;3:97–111.

[27] Ziemann U, Ilić TV, Iliać TV, et al Learning modifies subsequent induction of long-term potentiation-like and long-term depression-like plasticity in human motor cortex. J Neurosci 2004;24:1666–72. 10.1523/JNEUROSCI.5016-03.2004 14973238 PMC6730462

[28] Glannon W Consent to deep brain stimulation for neurological and psychiatric disorders. J Clin Ethics 2010;21:104. 20866016

[29] Lipsman N, Giacobbe P, Bernstein M, et al Informed consent for clinical trials of deep brain stimulation in psychiatric disease: challenges and implications for trial design. J Med Ethics 2012;38:107–11. 10.1136/jme.2010.042002 21685148

[30] Pugh J, Tan J, Aziz T, et al The moral obligation to prioritize research into deep brain stimulation over brain lesioning procedures for severe enduring anorexia Nervosa. Front Psychiatry 2018;9 10.3389/fpsyt.2018.00523 PMC6212472 30416458

[31] Paul LA Transformative experience: Oxford University Press, 2016.

[32] Leentjens AF, Visser-Vandewalle V, Temel Y, et al [Manipulation of mental competence: an ethical problem in case of electrical stimulation of the subthalamic nucleus for severe Parkinson’s disease]. Ned Tijdschr Geneeskd 2004;148:1394–8. 15291423

[33] Wilson S, Bladin P, Saling M The "burden of normality": concepts of adjustment after surgery for seizures. J Neurol Neurosurg Psychiatry 2001;70:649–56. 10.1136/jnnp.70.5.649 11309460 PMC1737335

[34] Gilbert F The burden of normality: from ’chronically ill' to ’symptom free'. New ethical challenges for deep brain stimulation postoperative treatment. J Med Ethics 2012;38:408–12. 10.1136/medethics-2011-100044 22431560

